# High Expression Levels of SLC38A1 Are Correlated with Poor Prognosis and Defective Immune Infiltration in Hepatocellular Carcinoma

**DOI:** 10.1155/2021/5680968

**Published:** 2021-10-16

**Authors:** Yun Liu, Yong Yang, Linna Jiang, Hongrui Xu, Junwei Wei

**Affiliations:** ^1^Department of General Surgery, First Hospital of Handan City, Handan, Hebei 056000, China; ^2^Department of General Surgery, Cixian Cancer Hospital, Handan, Hebei 056000, China; ^3^Pathology Department, First Hospital of Handan City, Handan, Hebei 056000, China; ^4^Translational Medicine Center, Huaihe Hospital of Henan University, Kaifeng, Henan 475000, China; ^5^Department of Gastroenterology, First Hospital of Handan City, Handan, Hebei 056000, China; ^6^Department of Infectious Diseases, Third Affiliated Hospital of Hebei Medical University, Shijiazhuang, Hebei 050000, China

## Abstract

Solute Carrier Family 38 Member 1 (SLC38A1) is a principal transporter of glutamine and plays a crucial role in the transformation of neoplastic cells. However, the correlation between SLC38A1 expression, prognosis, and immune infiltration in hepatocellular carcinoma (HCC) has yet to be elucidated. We used two independent patient cohorts, namely, a *Cancer* Genome Atlas (TCGA) cohort and a Clinical Proteomic Tumor Analysis Consortium (CPTAC) cohort, to analyze the role of SLC38A1 in HCC at the mRNA and protein levels, respectively. In these two cohorts, SLC38A1 mRNA and protein expression levels were higher in HCC tissues than in adjacent nontumor tissues. Both SLC38A1 mRNA and protein expression were positively associated with clinicopathological characteristics (clinical stage, *T* stage, pathological grade, tumor size, and tumor thrombus), were negatively associated with survival, and were independent prognostic factors in HCC patients. Functional enrichment analyses further indicated that SLC38A1 was involved in multiple pathways related to amino acid metabolism, tumors, and immunity. High expression levels of SLC38A1 were inversely proportional to CD8+ T cells and directly proportional to macrophages M0, neutrophils, programmed cell death-1/programmed cell death ligand 1 (PD-1/PD-L1), and cytotoxic T lymphocyte-associated protein 4 (CTLA-4). Moreover, we used immunohistochemical analysis of tissue samples and other online databases to further validate the expression levels and prognostic significance of SLC38A1 in HCC. Collectively, our study demonstrated that the upregulated expression of SLC38A1 was related to an unfavorable prognosis and defective immune infiltration in HCC.

## 1. Introduction

Hepatocellular carcinoma (HCC) is the most common primary tumor of the liver and the fourth leading cause of global cancer-related deaths [1]. A previous study that was based on the Surveillance, Epidemiology, and End Results Program (SEER) registry project indicated that the incidence of HCC will continue to rise in the future and is expected to peak in the year 2030 [2]. While treatment strategies for liver cancer have expanded with the emergence of new therapies, the prognoses of patients in advanced stages of HCC are still relatively unsatisfactory [3]. Therefore, it is of great significance to identify an effective biomarker that could predict prognosis and could be used as a therapeutic target for HCC patients [4].

The Solute Carrier Family 38 (SLC38) is the principal transporter for glutamine and plays a major role in maintaining homeostasis in the body [5]. Glutamine has a large number of vital functions in mammalian cells; consequently, the dysregulation of the SLC38 transporter may result in tumorigenesis and the progression of cancers [6]. The SLC38 transporter has been described as having the functional nature and regulatory mode of system A and system N transport activities [7]. As the first member of system A, SLC38A1 is predominantly expressed in the placenta and the brain [8]. Like other family members, the main function of SLC38A1 is to regulate the transport of short chain neutral amino acids, including glutamine. Although the upregulation of SLC38A1 expression has been demonstrated in a variety of tumors [9–12], the prognostic significance of SLC38A1 expression has not been reported in HCC. Furthermore, immunotherapy has been considered as a promising treatment for cancers, including HCC. It is also possible that tumor-infiltrating lymphocytes may influence the efficacy of immunotherapy. However, the correlation between SLC38A1 expression and immune infiltration in HCC has yet to be determined.

In the present study, we used various public databases to comprehensively analyze the expression of SLC38A1 and evaluate its prognostic significance at the mRNA and protein levels. We further validated the differential expression levels of SLC38A1 between HCC and adjacent nontumor tissue by performing immunohistochemistry (IHC) on our tissue samples. We also performed multiple enrichment analysis to explore the potential molecular mechanisms that might be mediated by SLC38A1 in HCC. In addition, we investigated the correlations between SLC38A1 expression and tumor-infiltrating immune cells (TIICs) or immune checkpoints. These analyses demonstrated that SLC38A1 was expressed at higher levels in HCC and associated with an unfavorable prognosis and defective immune infiltration.

## 2. Materials and Methods

### 2.1. mRNA Expression and Clinical Data from the TCGA Database

We used The *Cancer* Genome Atlas (TCGA) database to investigate the mRNA expression patterns of SLC38A1 in HCC. We downloaded transcriptome and corresponding clinical data for HCC patients from the Genomic Data Commons Data Portal (https://portal.gdc.cancer.gov/). The TCGA RNAseq data consisted of 424 samples, with 374 samples of HCC tissues and 50 samples of adjacent hepatic tissues. We selected HCC samples with a complete set of clinical information for the subsequent analysis of clinical significance. The clinicopathological characteristics included age, sex, pathological grade, clinical stage, tumor stage (T), lymphatic metastasis (N), distant metastasis (M), survival time, and survival status.

### 2.2. Protein Expression and Clinical Data from the CPTAC Database

We used the Clinical Proteomic Tumor Analysis Consortium (CPTAC) database to explore the protein expression profiles of SLC38A1 in HCC. The CPTAC database is a centralized data repository of proteomics data sets and clinical data for a variety of cancers (https://proteomics.cancer.gov/data-portal). We downloaded and extracted protein expression data relating to SLC38A1 and corresponding clinical data from the CPTAC-HCC proteome, including 159 samples of tumor tissue and adjacent hepatic tissue. The unshared log-ratio value was defined as the protein expression value. The clinicopathological characteristics included age, sex, tumor differentiation, medical history of liver cirrhosis, tumor size, tumor thrombus, tumor encapsulation, and survival time.

### 2.3. The Analysis of Other Online Databases

The Oncomine database combines 715 datasets and 86733 samples into one comprehensive database that aims to help researchers design better experiments and obtain more robust results (https://www.oncomine.org/) [13]. We performed meta-analysis of SLC38A1 RNA expression using the Oncomine database with a threshold of *p* ≤ 0.01 and a fold change (FC) ≥ 1.5. The Hepatocellular Carcinoma Expression Atlas Database (HCCDB) is an online resource for comprehensively annotating liver cancer gene expression and includes 15 public HCC expression datasets from 4000 samples (http://lifeome.net/database/hccdb/home.html) [14]. We used this database to further confirm whether the mRNA expression of SLC38A1 in HCC tissues was higher than that in nontumor tissues. The Kaplan–Meier plotter database offers a convenient way to assess the impact of multiple genes on survival in patients with 21 different cancer types (http://kmplot.com/analysis/) [15]. In the present study, we used this database to validate the correlation of SLC38A1 expression with the prognosis of HCC patients.

### 2.4. Immunohistochemistry

We retrospectively collected 30 pairs of paraffin-embedded HCC tissues and adjacent nontumor tissue samples from surgeries taking place between May 2019 and December 2020 at the Third Hospital of Hebei Medical University. This study was approved by the Ethics Committee of the Third Hospital of Hebei Medical University and carried out in accordance with the principles of the Declaration of Helsinki. The need for informed consent was waived by the Ethics Committee of the Third Hospital of Hebei Medical University given the retrospective nature of the study.

The paraffin-embedded tissue samples were sectioned, deparaffinized, hydrated, and boiled in a pressure cooker for antigen retrieval. The sections were then incubated with 3% hydrogen peroxide to inactivate endogenous peroxidase activity. Next, the samples were blocked in 10% goat serum and incubated overnight with a rabbit anti-human primary SLC38A1 antibody (Proteintech, China) at 4°C. Next, the sections were incubated with goat anti-rabbit horseradish peroxidase (HRP) conjugated secondary antibody (ZSGB-Bio, China) at 37°C. Finally, the sections were incubated with 3,3′-diaminobenzidine (DAB) and stained with hematoxylin. Two experienced pathologists independently assessed the samples. Each tissue sample was scored according to the intensity of staining and the proportion (%) of tumor cells that were stained. The scores ranged from 0 to 3; a score of 0–1 was considered as a negative stain while a score of 2–3 was considered as a positive stain.

### 2.5. Gene Set Enrichment Analysis (GSEA)

We used GSEA software (version 4.1.0) to explore the potential signaling pathways by which SLC38A1 may be involved in HCC. In this study, we categorized samples as either high- or low-SLC38A1 phenotypes in accordance with the median value of SLC38A1 expression from the TCGA database. Annotated gene sets (c2.cp.kegg.v7.3.symbols.gmt) were used as internal gene sets. The phenotypic enrichment pathways were sorted based on the nominal *p* value and normalized enrichment score (NES) [16]. Nominal *p* value and false discovery rate (FDR) q-value less than 0.05 were considered to be statistically significant.

### 2.6. Gene Coexpression and GO/KEGG Enrichment Analysis

We screened the genes that were coexpressed with SLC38A1 from the CPTAC database. Those with an absolute value of Pearson's correlation coefficient |R| ≥ 0.4 and a *p* value < 0.001 were selected as the screening threshold. Then, we performed Gene Ontology (GO) and Kyoto Encyclopedia of Genes and Genomes (KEGG) enrichment analysis of the coexpression genes by the *R* package “clusterProfiler” (version 3.18.1) which used multiple databases, including Disease Ontology database, Network of *Cancer* Gene database, Gene Ontology database, KEGG database, and Reactome Pathway database [17]. Before performing enrichment analysis, gene symbol codes were converted to Entrez ID by using human genome annotation package “org.Hs.eg.db.” Adjusted *p* values that were <0.05 were considered to be statistically significant.

### 2.7. Analysis of the Immune Landscape Related to the Expression Level of SLC38A1

CIBERSORT is a tool for deconvolving the expression matrix of immune cell subtypes based on the principle of linear support vector regression (http://cibersort.stanford.edu/) [18]. Using CIBERSORT analysis, it is possible to estimate the proportion of tumor-infiltrating immune cells (TIICs). In this study, we calculated the proportion of 22 TIICs in all HCC samples from the TCGA dataset. Tumor samples with a *p* value < 0.05 were chosen for subsequent analysis. Then, we carried out differential and correlation analysis to evaluate the correlation between SLC38A1 expression and TIICs or immune checkpoints. In the differential analysis, we divided the samples into high- and low-SLC38A1 expression groups in line with the median level of SLC38A1 expression. For the correlation analysis, our screening criteria were an |R| ≥ 0 and *p* value < 0.05.

### 2.8. Statistical Analysis

Statistical analyses were performed on the IHC data using SPSS software (version 23.0) and McNemar's test. All statistical analyses from the TCGA and CPTAC datasets were performed with R software (version 4.0.3). The median level of SLC38A1 expression was considered as a cutoff value. The association of clinicopathological characteristics with SLC38A1 expression was analyzed by Wilcoxon's rank sum test or the Kruskal–Wallis rank sum test and logistic regression. The effect of SLC38A1 expression on survival was evaluated by the Kaplan–Meier method, followed by the log-rank test. The effects of SLC38A1 expression and other clinicopathological characteristics on survival were compared by using univariate and multivariate Cox regression. Receiver Operating Characteristic (ROC) curves and the area under the curve (AUC) were used to compare the predictive accuracy of SLC38A1 expression with other clinicopathological characteristics. Correlation analysis of gene expression was evaluated by Spearman's correlation coefficient and statistical significance. A *p* value or FDR <0.05 was considered to be statistically significant.

## 3. Results

### 3.1. The Expression Levels of SLC38A1 Were Upregulated in HCC

**Figure 1:**
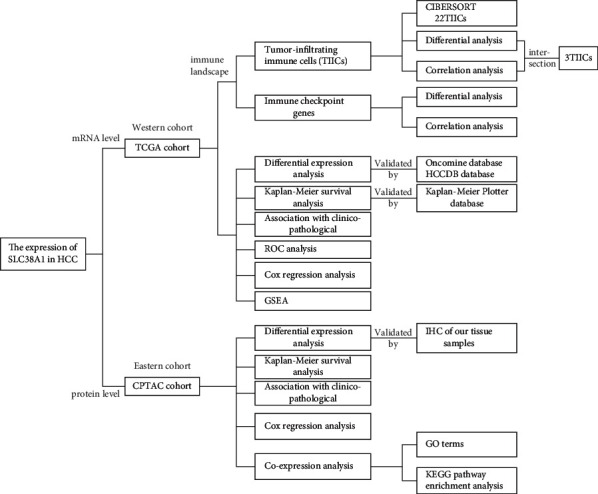
Analytical workflow of this study. GSEA, gene set enrichment analysis; GO, gene ontology; HCC, hepatocellular carcinoma; IHC, immunohistochemistry; KEGG, Kyoto Encyclopedia of Genes and Genomes; ROC, receiver operating characteristic.

**Figure 2:**
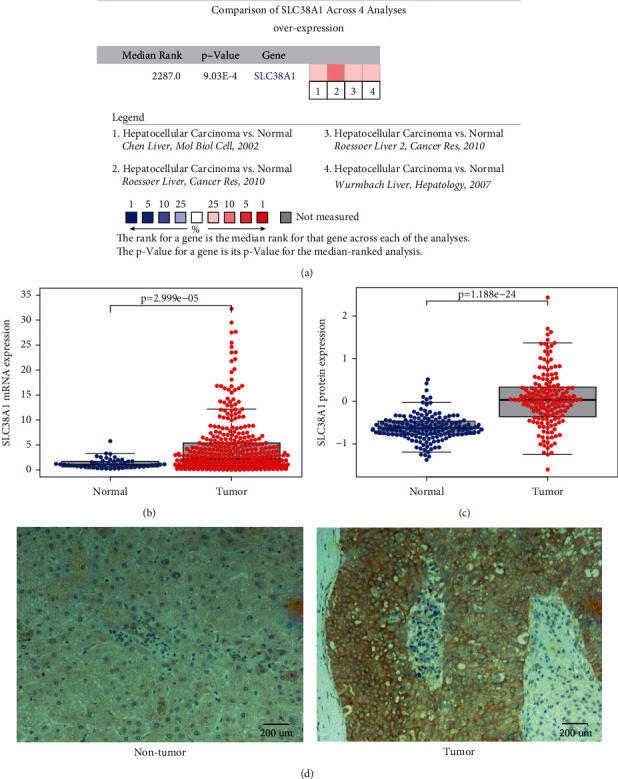
The expression levels of SLC38A1 in HCC. (a) Meta-analysis of SLC38A1 expression levels in HCC tissues relative to nontumor tissues, as determined by the Oncomine database. (b) Comparison of SLC38A1 mRNA expression level in HCC tissues and adjacent nontumor tissue, as determined by the TCGA database. (c) Comparison of SLC38A1 protein expression levels in HCC tissues and adjacent nontumor tissues, as determined by the CPTAC database. (d) Representative immunohistochemical images of SLC38A1 protein expression in adjacent nontumor tissues and HCC tissues from our hospital (scale bars = 200 *μ*m).

The analytical process used in the present study is shown in Figure 1. We first performed a meta-analysis of SLC38A1 mRNA levels from the Oncomine database which included four HCC studies that used the same thresholds as described above [19–21]. Our analyses demonstrated that the mRNA expression of SLC38A1 in the tumor group was significantly higher than that in the nontumor group (Figure 2(a)). Then, we analyzed the mRNA and protein expression of SLC38A1 from the TCGA and CPTAC databases, respectively. Our results showed that both the mRNA and protein expression of SLC38A1 was upregulated in tumor samples when compared to adjacent nontumor samples (Figures 2(b) and 2(c)). Furthermore, the online HCCDB database showed consistent results based on data from the Gene Expression Omnibus (GEO) and the International *Cancer* Genome Consortium (ICGC) datasets. Further details were given in Supplementary Table 1. Moreover, the IHC staining results from 30 paired HCC tissues in our hospital further confirmed the significantly higher protein levels of SLC38A1 in tumor tissues (76.7% vs. 46.7%, *p* < 0.05). Representative immunohistochemical images of SLC38A1 protein expression are shown in Figure 2(d). Collectively, these data indicated that SLC38A1 expression was upregulated in HCC.

### 3.2. The Upregulation of SLC38A1 Expression Predicted a Worse Prognosis in HCC Patients

**Figure 3:**
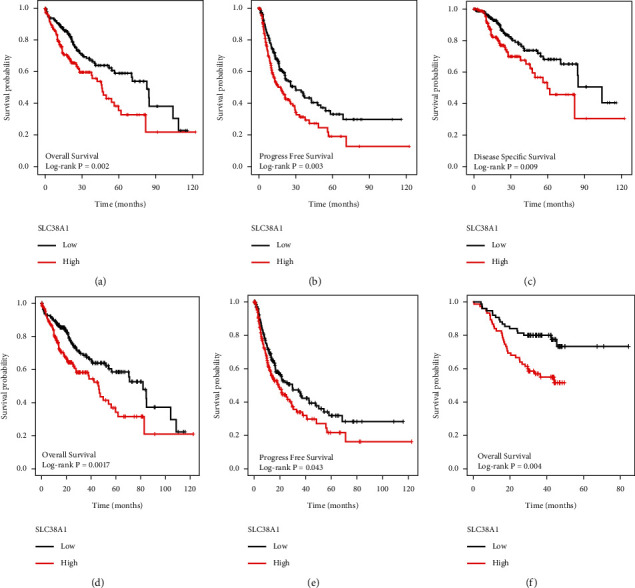
The effect of SLC38A1 expression on survival in HCC patients. (a) OS in TCGA. (b) PFS in TCGA. (c) DSS in TCGA. (d) OS in K-M plotter. (e) PFS in K-M plotter. (f) OS in CPTAC. OS, overall survival; PFS, progression-free survival; DSS, disease-specific survival.

First, we evaluated the impact of SLC38A1 expression on survival using the TCGA dataset. We found that high expression levels of SLC38A1 were significantly associated with poor overall survival (OS), progression-free survival (PFS), and disease-specific survival (DSS) (*p* = 0.002*, p* = 0.003, *p* = 0.009, respectively; Figures 3(a) and 3(c)). Then, we used the online Kaplan–Meier plotter database to validate the prognostic value of SLC38A1 in HCC and obtained results that were consistent with the TCGA database (Figures 3(d) and 3(e)). We also used the CPTAC database to further verify the relationship between the expression level of SLC38A1 protein and prognosis; the higher the expression level of SLC38A1, the worse the prognosis for HCC patients (*p* = 0.004, Figure 3(f)).

### 3.3. The Expression Level of SLC38A1 Was Associated with the Clinicopathological Characteristics of HCC Patients

**Figure 4:**
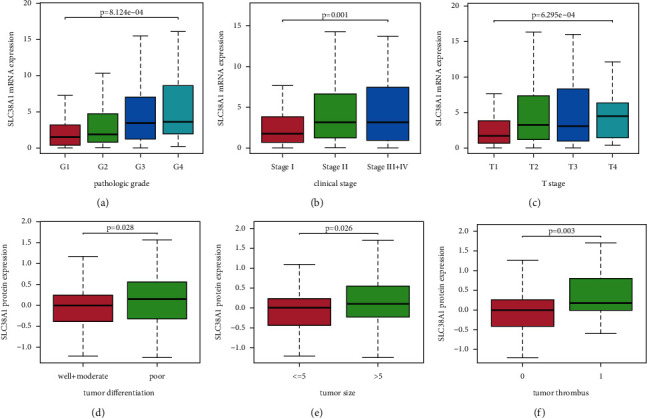
The association between SLC38A1 expression and clinicopathological characteristics. (a) Pathological grade, as determined from the TCGA database. (b) Clinical stage, as determined from the TCGA database. (c) T stage, as determined from the TCGA database. (d) Tumor differentiation, as determined from the CPTAC database. (e) Tumor size, as determined from the CPTAC database. (f) Tumor thrombus, as determined from the CPTAC database. The number of patients in the TCGA database that were in stage IV was very small; thus, patients with stages III and IV were pooled together for analysis. There was one patient in the well differentiation group derived from the CPTAC database; therefore, patients in the well differentiation and moderate differentiation groups were pooled for analysis.

As shown in Figures 4(a) and 4(c), the upregulated mRNA expression of SLC38A1 was significantly correlated with the pathological grade of tumors (*p* = 8.124E-04), clinical stage (*p* = 0.001), and *T* stage (*p* = 6.295E-04), based on the TCGA database. Figures 4(d) and 4(f) show that high protein levels of SLC38A1 were significantly associated with tumor differentiation (*p* = 0.028), tumor size (*p* = 0.026), and tumor thrombus (*p* = 0.003), as based on the CPTAC database. Univariate logistic regression analysis further showed that the upregulation of SLC38A1 expression in HCC was significantly correlated with high pathological grade, clinical stage, *T* stage, tumor differentiation, and tumor thrombus (Tables 1 and 2). These results further revealed that the upregulation of SLC38A1 expression was significantly associated with poor clinicopathological characteristics and suggested that HCC patients with high levels of SLC38A1 expression are more likely to progress to advanced stages than those with low levels of SLC38A1 expression.

### 3.4. SLC38A1 Represents an Independent Prognostic Predictor for Patients with HCC

**Figure 5:**
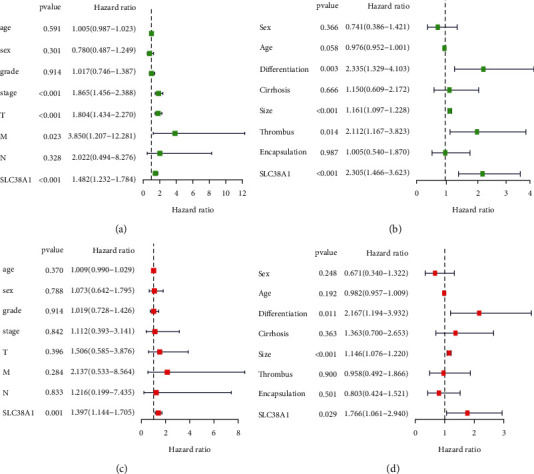
Univariate and multivariate Cox regression analysis of SLC38A1 expression and other clinicopathological characteristics. (a) Univariate cox regression analysis, as based on the TCGA database. (b) Univariate cox regression analysis, as based on the CPTAC database. (c) Multivariate cox regression analysis, as based on the TCGA database. (d) Multivariate cox regression analysis, as based on the CPTAC database.

**Figure 6:**
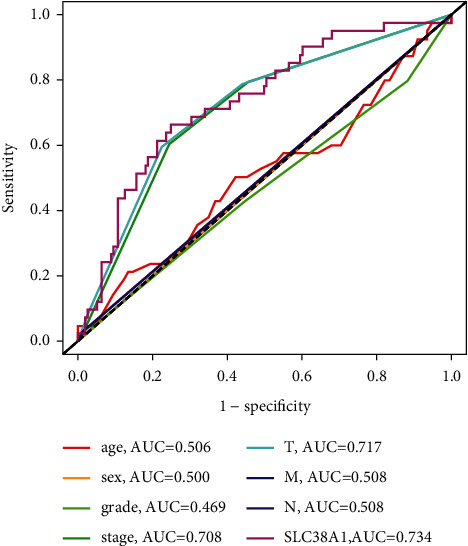
The AUCs of a range of prognostic predictors (including age, sex, grade, tumor stage, T stage, M stage, N stage, and SLC38A1). AUC, area under the curve.

As shown in Figures 5(a) and 5(b), the univariate cox regression analysis showed that high levels of SLC38A1 expression were significantly associated with a poor OS (hazard ration [HR]: 1.482, 95% confidence interval [CI]: 1.232–1.784, *P* < 0.001; HR: 2.305, 95% CI:1.466–3.623, *P* < 0.001, from the TCGA and CPTAC databases, respectively). Although other clinicopathological characteristics including clinical stage, *T* stage, *M* stage, differentiation, tumor size, and tumor thrombus, were also related to OS, as based on univariate cox regression analysis, only the expression levels of SLC38A1 remained associated with OS when we performed multivariate cox regression analysis (HR: 1.397, 95% CI: 1.144–1.705, *P* < 0.001; HR: 1.766, 95%CI: 1.061–2.940, *P* = 0.029, respectively), as shown in Figures 5(c) and 5(d). Therefore, our univariate and multivariate Cox regression analysis demonstrated that the expression level of SLC38A1 was an independent prognostic factor for HCC patients. To further evaluate the predictive accuracy of SLC38A1 expression, we performed ROC. Compared to other predictive factors (age, sex, tumor pathological grade, clinical stage, *T* stage, N stage and *M* stage), the AUC for SLC38A1 expression was greater (AUC = 0.734, Figure 6).

### 3.5. The Potential Molecular Mechanisms That Might Be Mediated by SLC38A1 in HCC

#### 3.5.1. GSEA

**Figure 7:**
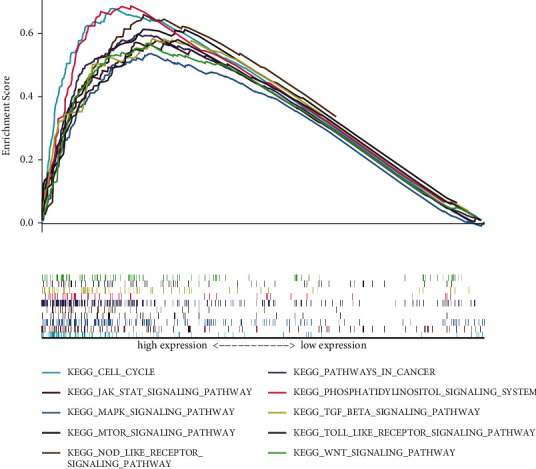
GSEA analysis of KEGG signaling pathways activated by high expression of SLC38A1 in HCC. GSEA, gene set enrichment analysis; KEGG, Kyoto Encyclopedia of Genes and Genomes.

We performed GSEA between high- and low-SLC38A1 expression phenotypes to explore potential signaling pathways based on the TCGA dataset. According to NES and FDR values, we selected significantly enriched KEGG signaling pathways. We identified a total of 85 signaling pathways that were differentially enriched in the high expression SLC38A1 phenotype (Supplementary Table 2). The most typically enriched signaling pathways are shown in Figure 7; analysis showed that multiple pathways that are related to tumor and immunity were differentially enriched in the high expression SLC38A1 phenotype.

#### 3.5.2. Gene Coexpression and GO/KEGG Enrichment Analysis

**Figure 8:**
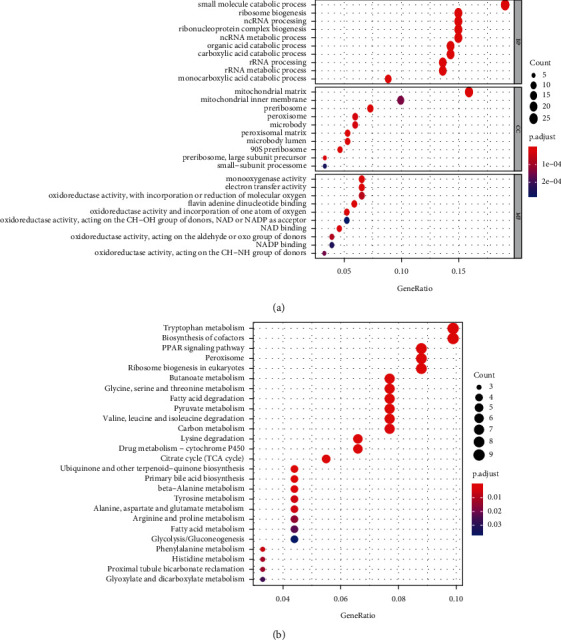
The top 10 GO terms and KEGG pathways related to coexpressed genes with SLC38A1. (a) GO terms (including BP, CC, and MF). (b) KEGG pathways. BP, biological process; CC, cellular component; MF, molecular function; GO, gene ontology; KEGG, Kyoto Encyclopedia of Genes and Genomes.

To further explore the potential mechanisms that might be mediated by SLC38A1 in HCC at the protein level, we conducted coexpression and GO/KEGG enrichment analysis, as based on the CPTAC database. According to a threshold set by |R| and *p* values, a total of 158 genes were found to be coexpressed with SLC38A1 and selected for subsequent GO/KEGG enrichment analysis (Supplementary Table 3). GO annotation revealed that 142 biological processes (BP), 48 molecular functions (MF), and 19 cellular component (CC) terms were significantly enriched (adjusted *p* value < 0.05). The top 10 GO terms are shown in Figure 8(a). These data suggested that the genes that were coexpressed with SLC38A1 may have a regulatory effect on HCC via mitochondrial matrix, pre-ribosome, oxidoreductase activity, and small molecule catabolic process. In addition, we identified 26 KEGG pathways that were significantly enriched (adjusted *p* value < 0.05), as shown in Figure 8(b). These highly enriched pathways included tryptophan metabolism, glycine, serine and threonine metabolism, valine, leucine and isoleucine degradation, and the PPAR signaling pathway.

### 3.6. The Expression of SLC38A1 Was Correlated with Tumor-Infiltrating Immune Cells (TIICs) and Immune Checkpoint Genes

**Figure 9:**
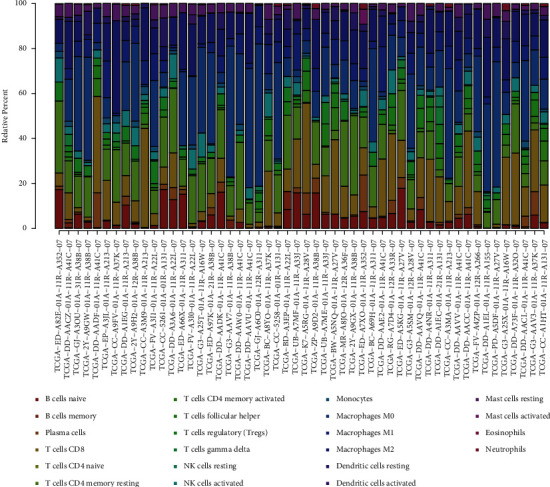
The proportion of 22 types of TIICs in HCC tumor samples. TIICs, tumor-infiltrating immune cells.

**Figure 10:**
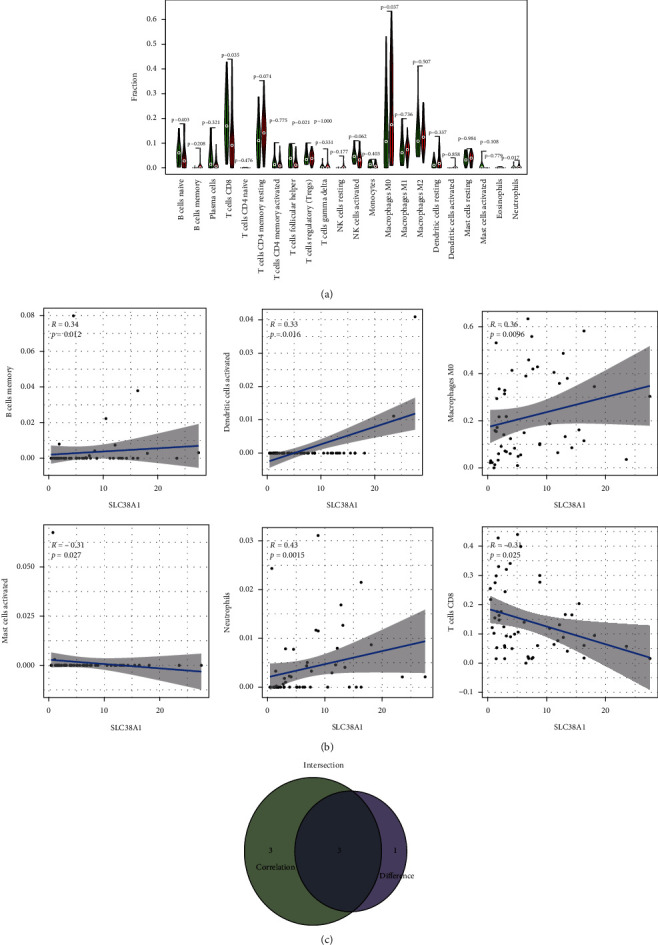
Correlation of SLC38A1 expression with TIICs. (a) Differential analysis of 22 TIICs between HCC tumor samples with high- and low-SLC38A1 expression levels. (b) Correlation analysis between SLC38A1 expression and the proportion of 6 TIICs (*p* < 0.05). (c) Venn diagrams showing 3 TIICs that were associated with the expression levels of SLC38A1, as determined by differential and correlation analysis. TIICs, tumor-infiltrating immune cells.

**Figure 11:**
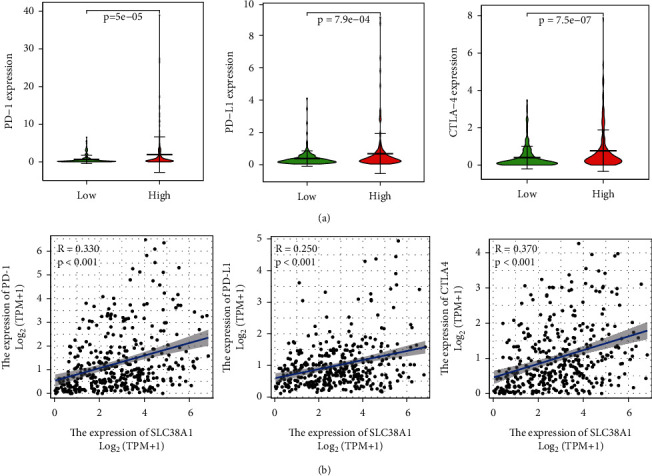
Correlation between SLC38A1 expression and immune checkpoint genes (PD-1, PD-L1, and CTLA-4). (a) Differential analysis of immune checkpoint genes with high and low expression levels of SLC38A1. (b) Correlation analysis between SLC38A1 expression and immune checkpoint genes.

To analyze the correlation between the expression levels of SLC38A1 and TIICs, we first calculated the proportion of 22 types of TIICs in HCC samples by CIBERSORT analysis (Figure 9). As shown in Figures 10(a) and 10(b), we obtained 4 and 6 types of TIICs that were significantly associated with SLC38A1 expression, as based on the differential and correlation analysis, respectively. As shown in Venn diagrams (Figure 10(c)), we obtained 3 types of TIICs (CD8+ T cell, Macrophages M0, and Neutrophils) that were associated with SLC38A1 expression, as determined by differential and correlation analysis. To be specific, high expression levels of SLC38A1 were inversely proportional to the numbers of CD8+ T cells and directly proportional to the numbers of macrophages M0 and neutrophils. Similarly, we used the differential analysis and correlation analysis to evaluate the relationships between SLC38A1 expression and immune checkpoints (PD-1, PD-L1, and CTLA-4). These analyses indicated that high expression levels of SLC38A1 were directly proportional to PD-1, PD-L1, and CTLA-4 (Figure 11).

## 4. Discussion

As a principal transporter of glutamine, SLC38A1 is selectively and physiologically expressed in normal human brain and placental tissues [8]. Studies have shown that SLC38A1 is overexpressed in malignant tumors and can promote the proliferative, invasive, and metastatic potentials of tumor cells [9–12]. However, prior to this study, the prognostic significance of SLC38A1 for patients with HCC was unknown. In the present study, we investigated the clinical significance of SLC38A1 by analyzing RNAseq and proteomic data. These public databases, along with IHC analysis of our own tissue samples, confirmed that the expression of SLC38A1 was upregulated in HCC. Both the mRNA and protein expression of SLC38A1 were associated with the clinicopathological characteristics and outcomes of HCC patients. In addition, we explored the correlations between SLC38A1 expression and TIICs or immune checkpoints; these data suggested that the upregulated expression of SLC38A1 was associated with defective immune infiltration in HCC.

In the present study, we combined results from public databases with our own data acquired from IHC of tissue samples and confirmed that the expression levels of SLC38A1 were upregulated in HCC; this was consistent with the findings of previous studies [9, 22]. However, neither of these studies evaluated the clinical and prognostic significance of SLC38A1 expression in HCC patients. In the present study, we found that the upregulation of SLC38A1 expression predicted a worse prognosis for HCC patients. Similarly, other studies have indicated that high levels of SLC38A1 expression represented an unfavorable prognostic indicator for human osteosarcoma, cholangiocarcinoma, gastric cancer, and acute myeloid leukemia [9–12, 23]. In addition, our study illustrated that the expression levels of SLC38A1 were positively associated with clinical stage, *T* stage, pathological grade, tumor size, and tumor thrombus, but were not significantly associated with *M* stage or N stage. Due to the small numbers of M1 and N1 patients in the TCGA database, it was not possible to define the precise association of SLC38A1 expression with lymphatic and distant metastasis. Therefore, it is necessary to perform further studies with larger sample sizes to confirm this finding. Moreover, our univariate and multivariate Cox regression analysis indicated that SLC38A1 expression was an independent predictor for prognosis in HCC patients. More importantly, the predictive accuracy of SLC38A1 expression was slightly better than that of the clinical stage. Collectively, these results strongly suggested that SLC38A1 expression is a good prognostic biomarker for HCC.

In order to further explore the potential mechanisms that might be mediated by SLC38A1 in HCC, we performed GSEA, coexpression analysis, and GO and KEGG enrichment analysis. GSEA results demonstrated that a phenotype characterized by high expression levels of SLC38A1 showed enrichment with tumor and immune-related pathways, including the JAK-STAT, Wnt, MAPK, mTOR ,and TGF-*β* signaling pathways. KEGG pathway enrichment analyses of genes that were coexpressed with SLC38A1 further indicated that SLC38A1 was involved in multiple metabolic pathways, the most important of which was amino acid metabolism; these findings were similar to those of a previous report.^5^ Therefore, we concluded that SLC38A1 is involved in pathways related to substance metabolism, tumors, and immunity. However, the specific regulatory mechanisms involved needs to be investigated further.

Another major finding of this study was that the expression levels of SLC38A1 correlated with TIICs in HCC. Previous studies have shown that tumor-infiltrating lymphocytes can serve as independent predictors of survival in cancer patients [24, 25]. For this reason, we explored the correlation between SLC38A1 expression and immune infiltration in HCC. It is well established that CD8+ T cells are the main effector cells against tumors and stimulate cell death via the Fas-Fas ligand pathway or by releasing perforin granules to eliminate tumors [26]. A recent study confirmed that the number of CD8+T cells is closely associated with the prognosis of patients with a variety of tumors [27]. Furthermore, recent studies have shown that neutrophils can stimulate tumor angiogenesis and mediate immune suppression mechanisms to promote tumor growth and metastasis, and also represents a biomarker of tumor prognosis [28, 29]. In the present study, we found that high expression levels of SLC38A1 were inversely proportional to CD8+ T cells and directly proportional to macrophages M0 and neutrophils; this explained why high levels of SLC38A1 can predict a poor prognosis, at least in part. A series of immune checkpoints, including CTLA-4, PD-1, PD-L1, and lymphocyte activation gene 3 (LAG-3), have been confirmed to be involved in the induction and maintenance of immune tolerance in HCC [30–32]. Moreover, immune checkpoint inhibitors have shown therapeutic potential for patients with advanced HCC in clinical trials [33, 34]. Therefore, we investigated the association between SLC38A1 expression and immune checkpoints and found that high expression levels of SLC38A1 were directly proportional to PD-1, PD-L1, and CTLA-4. This indicated that the poor outcomes of HCC patients with high levels of SLC38A1 expression may be attributable to the immunosuppressive microenvironment. Thus, we considered that SLC38A1 may have a potential impact on tumor immunology.

The main advantage of this study is that we comprehensively analyzed the prognostic significance of SLC38A1 expression at the RNA and protein levels; previous prognostic biomarkers based on bioinformatics mining were mainly focused on RNA levels. Furthermore, our study population included not only European and American populations but also Asian populations. Collectively, these factors increase the reliability and applicability of SLC38A1 as a prognostic biomarker for HCC patients. However, this study still has certain limitations that need to be considered. Firstly, there were clear differences in clinical characteristics between the TCGA and CPTAC databases. Secondly, the specific relationship between SLC38A1 expression and tumor immune infiltration requires further investigation.

## 5. Conclusions

In summary, we systematically analyzed the clinicopathological significance of SLC38A1 expression at the RNA and protein levels in HCC. Our study demonstrated that higher levels of SLC38A1 expression were associated with disease progression and a worse prognosis, as well as impaired immune infiltration in HCC.

## Figures and Tables

**Table 1:** The association between SLC38A1 expression and clinicopathological characteristics (logistic regression, TCGA database).

Clinicopathological characteristics	Total (*N*)	Odds ratio for SLC38A1 mRNA expression	*p* value
*Age (years)*			
>60 vs. ≤ 60	370	0.823 (0.546–1.237)	0.349

*Sex*			
Male vs. female	371	1.237 (0.801–1.915)	0.337

*Pathological grade*			
Grade II vs. grade I	232	1.527 (0.821–2.911)	0.187
Grade III vs. grade I	177	3.242 (1.683–6.411)	0.001^∗^
Grade IV vs. grade I	67	3.789 (1.053–15.750)	0.048^∗^

*Clinical stage*			
Stage II vs. stage I	257	2.104 (1.247–3.581)	0.006 ^*∗*^
Stage III + IV^a^ vs. stage I	261	2.380 (1.417–4.041)	0.001^∗^

Tumor stage (T)			
T2 vs. T1	275	2.185 (1.320–3.647)	0.003^∗^
T3 vs. T1	261	2.449 (1.435–4.233)	0.001^∗^
T4 vs. T1	194	3.486 (1.091–13.260)	0.044^∗^

*Lymphatic metastasis*			
Positive vs. negative	256	3.048 (0.384–62.071)	0.337

*Distant metastasis*			
Positive vs. negative	270	1.685E-07 (NA-2.860 E + 29)	0.983

*Note.* ^a^Since the number of patients with stage IV was very small, we pooled patients with stage III and stage IV for analysis.  ^*∗*^*p* < 0.05.

**Table 2:** The association between SLC38A1 expression and clinicopathological characteristics (logistic regression, CPTAC database).

Clinicopathological characteristics	Total (*N*)	Odds ratio for SLC38A1 protein expression	*p* value
*Age* (years)			
>60 vs. ≤ 60	152	0.523 (0.252–1.061)	0.076

*Sex*			
Male vs. female	152	1.181 (0.530–2.657)	0.684

*Differentiation*			
Poor vs. well + moderate^b^	152	2.619 (1.346–5.206)	0.005^∗^

*Cirrhosis*			
Positive vs. negative	152	1.284 (0.642–2.586)	0.480

*Size* (cm)			
>5 vs. ≤ 5	152	1.447 (0.765–2.753)	0.257

*Thrombus*			
Positive vs. negative	152	2.237 (1.051–4.935)	0.040^∗^

*Lymphatic metastasis*			
Positive vs. negative	152	1 (0.039–25.600)	1.000

*Encapsulation*			
Positive vs. negative	152	0.939 (0.466–1.887)	0.859

*Note.* ^b^There was only one patient in the well differentiation group. Therefore, patients with well and moderate differentiation were pooled for analysis;  ^*∗*^*p* < 0.05.

## Data Availability

The dataset of the public database can be found from the following websites: The *Cancer* Genome Atlas (TCGA) database: https://portal.gdc.cancer.gov/; The Clinical Proteomic Tumor Analysis Consortium (CPTAC) database: https://proteomics.cancer.gov/data-portal; The Oncomine database: https://www.oncomine.org/; The Hepatocellular Carcinoma Expression Atlas (HCCDB) database: http://lifeome.net/database/hccdb/home.html); and The Kaplan–Meier plotter database: http://kmplot.com/analysis/.

## Supplementary Materials

Supplementary Materials Supplementary Table 1: details of GEO series and ICGC dataset from the HCCDB database. Supplementary Table 2: gene sets enriched in phenotype high. Supplementary Table 3: coexpression genes of SLC38A1.
