# Supplementary material for: High Expression Levels of SLC38A1 Are Correlated with Poor Prognosis and Defective Immune Infiltration in Hepatocellular Carcinoma
Source: J Oncol. 2021 Oct 16;2021:5680968. doi: 10.1155/2021/5680968 (PMC8541878; doi:10.1155/2021/5680968)
Supplement: Supplementary Materials — Supplementary Table 1: details of GEO series and ICGC dataset from the HCCDB database. Supplementary Table 2: gene sets enriched in phenotype high. Supplementary Table 3: coexpression genes of SLC38A1. [file 5680968.f1.zip › 5680968.f1/Supplementary Table 1 (1).docx]

Supplementary Table 1 Details of GEO series and ICGC dataset from the HCCDB database

| Dataset ID | Type | Numbers | Mean | STD | IQR | p-value |
| --- | --- | --- | --- | --- | --- | --- |
| GSE25097 | HCC | 268 | 1.22 | 1.072 | 1.335 | 0.0000589 |
|  | Adjacent | 243 | 0.927 | 0.464 | 0.518 |  |
| GSE36376 | HCC | 240 | 6.7 | 0.6144 | 0.956 | 9.63E-27 |
|  | Adjacent | 193 | 6.197 | 0.2193 | 0.3119 |  |
| GSE14520 | HCC | 225 | 6.251 | 1.462 | 2.566 | 3.39E-14 |
|  | Adjacent | 220 | 5.386 | 0.7213 | 1.019 |  |
| GSE46444 | HCC | 88 | 6.657 | 0.5512 | 0.6751 | 0.004445 |
|  | Adjacent | 48 | 6.99 | 0.6751 | 0.8804 |  |
| GSE76427 | HCC | 115 | 7.731 | 0.6873 | 0.9825 | 0.001426 |
|  | Adjacent | 52 | 7.471 | 0.3476 | 0.3687 |  |
| ICGC | HCC | 212 | 2.104 | 1.24 | 1.988 | 0.006855 |
|  | Adjacent | 177 | 1.828 | 0.7284 | 0.98 |  |

GEO, Gene Expression Omnibus; ICGC, International Cancer Genome Consortium; STD, standard deviation; IQR, interquartile range.
