# Supplementary material for: High Expression Levels of SLC38A1 Are Correlated with Poor Prognosis and Defective Immune Infiltration in Hepatocellular Carcinoma
Source: J Oncol. 2021 Oct 16;2021:5680968. doi: 10.1155/2021/5680968 (PMC8541878; doi:10.1155/2021/5680968)
Supplement: Supplementary Materials — Supplementary Table 1: details of GEO series and ICGC dataset from the HCCDB database. Supplementary Table 2: gene sets enriched in phenotype high. Supplementary Table 3: coexpression genes of SLC38A1. [file 5680968.f1.zip › 5680968.f1/Supplementary Table 2 (1).docx]

Supplementary Table 2 Gene sets enriched in phenotype high

| Gene set name | NES | NOM  *p*-value | FDP  q-value |
| --- | --- | --- | --- |
| KEGG_Phosphatidylinositol signaling system | 2.07 | 0.000 | 0.012 |
| KEGG_Apoptosis | 2.06 | 0.000 | 0.007 |
| KEGG_Pathways in cancer | 2.06 | 0.000 | 0.004 |
| KEGG_FCγR_mediated phagocytosis | 2.05 | 0.000 | 0.004 |
| KEGG_Inositol phosphate metabolism | 2.04 | 0.000 | 0.003 |
| KEGG_Vasopressin regulated water reabsorption | 2.04 | 0.000 | 0.003 |
| KEGG_Endocytosis | 2.02 | 0.000 | 0.003 |
| KEGG_Small cell lung cancer | 2.02 | 0.000 | 0.003 |
| KEGG_Ubiquitin mediated proteolysis | 2.02 | 0.000 | 0.003 |
| KEGG_Oocyte meiosis | 2.01 | 0.000 | 0.003 |
| KEGG_Pancreatic cancer | 2.01 | 0.000 | 0.003 |
| KEGG_Regulation of actin cytoskeleton | 2.01 | 0.000 | 0.003 |
| KEGG_Renal cell carcinoma | 2.00 | 0.000 | 0.003 |
| KEGG_Chronic myeloid leukemia | 1.98 | 0.000 | 0.004 |
| KEGG_Nod like receptor signaling pathway | 1.97 | 0.002 | 0.004 |
| KEGG_JAK STAT signaling pathway | 1.95 | 0.000 | 0.005 |
| KEGG_Focal adhesion | 1.94 | 0.002 | 0.006 |
| KEGG_Epithelial cell signaling in helicobacter pylori infection | 1.94 | 0.000 | 0.006 |
| KEGG_RNA degradation | 1.94 | 0.002 | 0.006 |
| KEGG_Adherens junction | 1.93 | 0.000 | 0.006 |
| KEGG_Progesterone mediated oocyte maturation | 1.92 | 0.000 | 0.007 |
| KEGG_Rig I like receptor signaling pathway | 1.91 | 0.000 | 0.007 |
| KEGG_Colorectal cancer | 1.91 | 0.000 | 0.007 |
| KEGG_WNT signaling pathway | 1.90 | 0.000 | 0.007 |
| KEGG_Axon guidance | 1.90 | 0.000 | 0.007 |
| KEGG_Pathogenic Escherichia coli infection | 1.89 | 0.000 | 0.008 |
| KEGG_MAPK signaling pathway | 1.89 | 0.000 | 0.008 |
| KEGG_Cell cycle | 1.89 | 0.000 | 0.007 |
| KEGG_Purine metabolism | 1.89 | 0.000 | 0.007 |
| KEGG_Neurotrophin signaling pathway | 1.89 | 0.002 | 0.007 |
| KEGG_Prostate cancer | 1.89 | 0.002 | 0.007 |
| KEGG_Natural killer cell mediated cytotoxicity | 1.88 | 0.000 | 0.007 |
| KEGG_Toll like receptor signaling pathway | 1.87 | 0.000 | 0.008 |
| KEGG_P53 signaling pathway | 1.87 | 0.000 | 0.008 |
| KEGG_Leukocyte transendothelial migration | 1.87 | 0.000 | 0.008 |
| KEGG_ERBB signaling pathway | 1.86 | 0.000 | 0.008 |
| KEGG_T cell receptor signaling pathway | 1.86 | 0.000 | 0.008 |
| KEGG_Insulin signaling pathway | 1.86 | 0.002 | 0.008 |
| KEGG_mTOR signaling pathway | 1.85 | 0.000 | 0.009 |
| KEGG_Snare interactions in vesicular transport | 1.85 | 0.002 | 0.009 |
| KEGG_TGFβ signaling pathway | 1.84 | 0.004 | 0.010 |
| KEGG_Non-small cell lung cancer | 1.83 | 0.000 | 0.010 |
| KEGG_Chemokine signaling pathway | 1.83 | 0.002 | 0.010 |
| KEGG_Long term potentiation | 1.82 | 0.002 | 0.010 |
| KEGG_Glioma | 1.82 | 0.002 | 0.011 |
| KEGG_Pyrimiding metabolism | 1.81 | 0.004 | 0.012 |
| KEGG_Tight junction | 1.80 | 0.004 | 0.012 |
| KEGG_Bladder cancer | 1.80 | 0.000 | 0.013 |
| KEGG_Vibrio cholerae infection | 1.80 | 0.002 | 0.013 |
| KEGG_B cell receptor signaling pathway | 1.80 | 0.006 | 0.013 |
| KEGG_Gap junction | 1.79 | 0.006 | 0.013 |
| KEGG_Basal transcription factors | 1.79 | 0.002 | 0.013 |
| KEGG_Gnrh signaling pathway | 1.77 | 0.004 | 0.015 |
| KEGG_Cytokine cytokine receptor interaction | 1.77 | 0.010 | 0.015 |
| KEGG_Cytosolic DNA sensing pathway | 1.77 | 0.006 | 0.015 |
| KEGG_Homologous recombination | 1.77 | 0.000 | 0.015 |
| KEGG_VEGF signaling pathway | 1.76 | 0.002 | 0.016 |
| KEGG_Notch signaling pathway | 1.75 | 0.006 | 0.018 |
| KEGG_Mismatch repair | 1.74 | 0.004 | 0.019 |
| KEGG_Melanogenesis | 1.74 | 0.004 | 0.019 |
| KEGG_Spliceosome | 1.74 | 0.008 | 0.019 |
| KEGG_Arrhythmogenic right ventricular cardiomyopathy arvc | 1.73 | 0.006 | 0.020 |
| KEGG_Hypertrophic cardiomyopathy hcm | 1.73 | 0.006 | 0.021 |
| KEGG_Ecm receptor interaction | 1.72 | 0.024 | 0.022 |
| KEGG_Acute myeloid leukemia | 1.72 | 0.004 | 0.021 |
| KEGG_Endometrial cancer | 1.71 | 0.016 | 0.023 |
| KEGG_Leishmania infection | 1.71 | 0.025 | 0.023 |
| KEGG_Long term depression | 1.70 | 0.010 | 0.024 |
| KEGG_Dorso ventral axis formation | 1.69 | 0.010 | 0.026 |
| KEGG_Type II diabetes mellitus | 1.69 | 0.002 | 0.026 |
| KEGG_Melanoma | 1.69 | 0.006 | 0.026 |
| KEGG_FC epsilon ri signaling pathway | 1.69 | 0.010 | 0.026 |
| KEGG_Glycosphingolipid biosynthesis lacto and neolacto series | 1.66 | 0.012 | 0.032 |
| KEGG_Glycerophospholipid metabolism | 1.66 | 0.006 | 0.033 |
| KEGG_N Glycan biosynthesis | 1.66 | 0.016 | 0.033 |
| KEGG_Dilated cardiomyopathy | 1.65 | 0.010 | 0.034 |
| KEGG_Nucleotide excision repair | 1.64 | 0.031 | 0.036 |
| KEGG_Selenoamino acid metabolism | 1.64 | 0.030 | 0.039 |
| KEGG_Viral myocarditis | 1.62 | 0.035 | 0.042 |
| KEGG_Hedgehog signaling pathway | 1.62 | 0.022 | 0.042 |
| KEGG_Amyotrophic lateral sclerosis als | 1.61 | 0.004 | 0.046 |
| KEGG_Riboflavin metabolism | 1.61 | 0.018 | 0.045 |
| KEGG_DNA replication | 1.61 | 0.039 | 0.045 |
| KEGG_Basal cell carcinoma | 1.61 | 0.014 | 0.045 |
| KEGG_Glycosaminoglycan biosynthesis chondroitin sulfate | 1.60 | 0.022 | 0.048 |

NES: normalized enrichment score; NOM: nominal; FDR: false discovery rate; Gene sets with NOM *p*-value <0.05 and FDR q-value <0.05 were considered as significant.
