# Supplementary material for: High Expression Levels of SLC38A1 Are Correlated with Poor Prognosis and Defective Immune Infiltration in Hepatocellular Carcinoma
Source: J Oncol. 2021 Oct 16;2021:5680968. doi: 10.1155/2021/5680968 (PMC8541878; doi:10.1155/2021/5680968)
Supplement: Supplementary Materials — Supplementary Table 1: details of GEO series and ICGC dataset from the HCCDB database. Supplementary Table 2: gene sets enriched in phenotype high. Supplementary Table 3: coexpression genes of SLC38A1. [file 5680968.f1.zip › 5680968.f1/Supplementary Table 3 (1).docx]

Supplementary Table 3 Co-expression genes of SLC38A1

| Co-expression gene | Cor | *p*-value | Co-expression gene | Cor | *p*-value |
| --- | --- | --- | --- | --- | --- |
| ABAT | -0.448 | 3.27E-09 | MAOB | -0.452 | 2.16E-09 |
| ACBD4 | -0.401 | 1.64E-07 | 2-Mar | -0.473 | 3.07E-10 |
| ACOX2 | -0.457 | 1.40E-09 | MARCKS | 0.425 | 2.37E-08 |
| ACSM2A | -0.436 | 8.94E-09 | METTL26 | -0.442 | 5.59E-09 |
| AFG1L | -0.412 | 6.77E-08 | MLX | -0.402 | 1.49E-07 |
| ALDH2 | -0.458 | 1.26E-09 | MRPS36 | -0.404 | 1.33E-07 |
| ALDH7A1 | -0.466 | 5.82E-10 | MRTO4 | 0.45 | 2.67E-09 |
| APOC3 | -0.471 | 3.57E-10 | MSRB2 | -0.412 | 6.61E-08 |
| ARHGEF2 | 0.406 | 1.12E-07 | MTM1 | -0.425 | 2.28E-08 |
| ABHD14B | -0.425 | 2.42E-08 | MYBBP1A | 0.405 | 1.20E-07 |
| ACOT13 | -0.411 | 7.24E-08 | NAA25 | 0.407 | 9.78E-08 |
| ACSL3 | 0.447 | 3.59E-09 | NAP1L1 | 0.413 | 6.55E-08 |
| ACSM2B | -0.471 | 3.63E-10 | NAT10 | 0.424 | 2.47E-08 |
| ALAD | -0.482 | 1.27E-10 | NCL | 0.428 | 1.88E-08 |
| ALDH5A1 | -0.459 | 1.16E-09 | NIFK | 0.438 | 7.46E-09 |
| ANXA9 | -0.425 | 2.42E-08 | NIP7 | 0.418 | 4.33E-08 |
| ATP1B3 | 0.456 | 1.57E-09 | NIT1 | -0.464 | 7.13E-10 |
| BAZ1A | 0.412 | 6.81E-08 | NIT2 | -0.412 | 6.84E-08 |
| BCAT1 | 0.456 | 1.48E-09 | NOL11 | 0.476 | 2.36E-10 |
| BDH1 | -0.41 | 7.73E-08 | NOP2 | 0.423 | 2.68E-08 |
| BHMT2 | -0.425 | 2.34E-08 | NUDT6 | -0.404 | 1.25E-07 |
| BPHL | -0.533 | 4.82E-13 | OGDHL | -0.412 | 6.96E-08 |
| C11orf54 | -0.466 | 6.25E-10 | OSBPL3 | 0.414 | 5.90E-08 |
| CAMP | 0.42 | 3.67E-08 | PCK2 | -0.486 | 8.46E-11 |
| CAT | -0.431 | 1.37E-08 | PEBP1 | -0.477 | 2.10E-10 |
| CCDC88A | 0.424 | 2.51E-08 | PES1 | 0.442 | 5.54E-09 |
| CCL20 | 0.471 | 3.80E-10 | PIPOX | -0.48 | 1.61E-10 |
| CD3EAP | 0.407 | 1.04E-07 | PKM | 0.452 | 2.14E-09 |
| CMSS1 | 0.423 | 2.87E-08 | POLR1E | 0.468 | 5.16E-10 |
| COQ5 | -0.484 | 1.06E-10 | PPAN-P2RY11 | 0.416 | 4.75E-08 |
| COQ6 | -0.414 | 5.97E-08 | PPL | -0.414 | 5.81E-08 |
| COQ7 | -0.416 | 5.00E-08 | PRMT1 | 0.526 | 1.08E-12 |
| COQ9 | -0.417 | 4.39E-08 | PRNP | 0.441 | 5.74E-09 |
| CYB5A | -0.484 | 9.98E-11 | PUM3 | 0.478 | 1.89E-10 |
| CYP27A1 | -0.411 | 7.58E-08 | PUS7 | 0.407 | 1.03E-07 |
| CYP2C9 | -0.46 | 1.07E-09 | PYGL | -0.407 | 1.05E-07 |
| CYP2D6 | -0.464 | 7.11E-10 | PYURF | -0.416 | 4.94E-08 |
| CYP4F3 | -0.445 | 4.12E-09 | QDPR | -0.429 | 1.74E-08 |
| CYP8B1 | -0.474 | 2.84E-10 | RBM19 | 0.523 | 1.50E-12 |
| DAO | -0.427 | 1.94E-08 | RBP5 | -0.406 | 1.11E-07 |
| DCXR | -0.411 | 7.51E-08 | REPS2 | -0.438 | 7.91E-09 |
| DDX18 | 0.419 | 3.92E-08 | RNASE1 | 0.421 | 3.41E-08 |
| DDX21 | 0.465 | 6.68E-10 | RRP9 | 0.408 | 9.75E-08 |
| DHRS4 | -0.423 | 2.79E-08 | RSL1D1 | 0.405 | 1.20E-07 |
| DHTKD1 | -0.411 | 7.49E-08 | RTP3 | -0.504 | 1.26E-11 |
| DMGDH | -0.411 | 7.26E-08 | S100A8 | 0.408 | 9.39E-08 |
| DNAJC10 | 0.479 | 1.75E-10 | SAE1 | 0.403 | 1.36E-07 |
| EBNA1BP2 | 0.426 | 2.23E-08 | SDHA | -0.418 | 4.17E-08 |
| ECHDC2 | -0.401 | 1.59E-07 | SDHB | -0.426 | 2.09E-08 |
| ECI1 | -0.451 | 2.50E-09 | SEC14L2 | -0.423 | 2.85E-08 |
| ECI2 | -0.479 | 1.68E-10 | SELENBP1 | -0.469 | 4.54E-10 |
| EHHADH | -0.459 | 1.17E-09 | SERPINE1 | 0.498 | 2.34E-11 |
| ETFDH | -0.408 | 9.25E-08 | SGTB | 0.405 | 1.17E-07 |
| FAHD1 | -0.413 | 6.45E-08 | SLC16A3 | 0.403 | 1.35E-07 |
| FDX1 | -0.436 | 9.29E-09 | SLC1A5 | 0.49 | 5.47E-11 |
| FDXR | -0.445 | 4.07E-09 | SLC25A10 | -0.402 | 1.52E-07 |
| FH | -0.417 | 4.46E-08 | SLC25A20 | -0.407 | 1.04E-07 |
| FMO3 | -0.407 | 1.03E-07 | SLC25A42 | -0.447 | 3.44E-09 |
| FMO4 | -0.45 | 2.74E-09 | SLC27A5 | -0.433 | 1.20E-08 |
| FMO5 | -0.408 | 9.70E-08 | SLC38A1 | 1 | 0 |
| GALE | -0.407 | 1.04E-07 | SLC38A2 | 0.421 | 3.30E-08 |
| GATD3A | -0.409 | 8.36E-08 | SLC3A2 | 0.418 | 4.12E-08 |
| GLYCTK | -0.432 | 1.31E-08 | SLCO2B1 | -0.41 | 7.88E-08 |
| GPATCH4 | 0.409 | 8.91E-08 | SORBS2 | -0.452 | 2.15E-09 |
| GRHPR | -0.453 | 2.03E-09 | SORD | -0.456 | 1.59E-09 |
| GTPBP4 | 0.436 | 9.48E-09 | SPG7 | -0.421 | 3.21E-08 |
| HAAO | -0.412 | 6.94E-08 | SPP1 | 0.457 | 1.47E-09 |
| HADH | -0.449 | 2.85E-09 | SSB | 0.402 | 1.54E-07 |
| HAGH | -0.442 | 5.34E-09 | THNSL1 | -0.444 | 4.48E-09 |
| HDHD3 | -0.442 | 5.32E-09 | TLE3 | 0.408 | 9.18E-08 |
| HEATR1 | 0.415 | 5.33E-08 | TOP1 | 0.456 | 1.53E-09 |
| HINT2 | -0.426 | 2.08E-08 | UTP15 | 0.411 | 7.41E-08 |
| HPD | -0.426 | 2.22E-08 | UTP18 | 0.435 | 9.99E-09 |
| HSD17B8 | -0.405 | 1.22E-07 | UTP20 | 0.404 | 1.33E-07 |
| IBA57 | -0.419 | 4.01E-08 | UTP4 | 0.458 | 1.22E-09 |
| IGSF8 | -0.459 | 1.21E-09 | WDR12 | 0.456 | 1.51E-09 |
| IL4I1 | 0.439 | 7.24E-09 | WDR43 | 0.444 | 4.50E-09 |
| KIAA0930 | 0.469 | 4.37E-10 | WDR75 | 0.499 | 2.25E-11 |
| LPCAT1 | 0.435 | 1.01E-08 |  |  |  |

Cor: correlation coefficient.
